# 3,3,6,6-Tetra­methyl-9-[6-(3,3,6,6-tetra­methyl-1,8-dioxo-2,3,4,5,6,7,8,9-octa­hydro-1*H*-xanthen-9-yl)pyridin-2-yl]-2,3,4,5,6,7,8,9-octa­hydro-1*H*-xanthene-1,8-dione

**DOI:** 10.1107/S1600536811007318

**Published:** 2011-03-05

**Authors:** Antar A. Abdelhamid, Shaaban Kamel Mohamed, Mirze A. Allahverdiyev, Atash V. Gurbanov, Seik Weng Ng

**Affiliations:** a Department of Organic Chemistry, Baku State University, Baku, Azerbaijan; b Chemistry & Environmental Science Division, School of Science, Manchester Metropolitan University, UK; c Department of Chemistry, University of Malaya, 50603 Kuala Lumpur, Malaysia

## Abstract

In the title mol­ecule, C_39_H_45_NO_6_, the two tetra­methyl­octa­hydroxanthen-1,8-dione substituents are arranged approximately parallel to each other and approximately perpendicular to the plane of the pyridine ring. The six-membered xanthene rings adopt flattened boat conformations with the O and methine C atoms deviating from the plane of the other four atoms.

## Related literature

For a related structure, see: Mohamed *et al.* (2011 ▶).
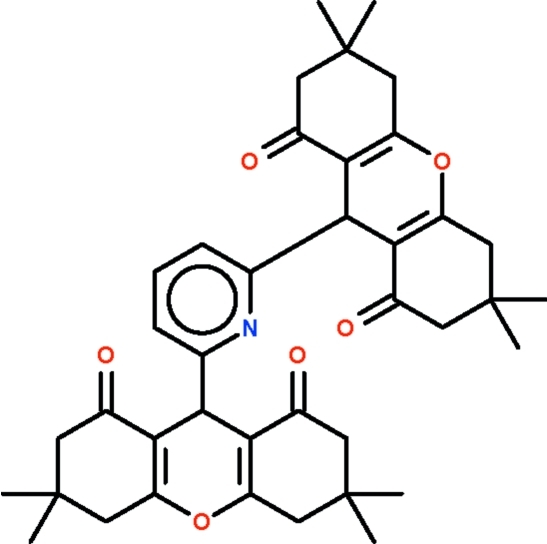


## Experimental

### 

#### Crystal data

C_39_H_45_NO_6_
*M* *_r_* = 623.76
Monoclinic, 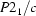

*a* = 24.1384 (8) Å
*b* = 10.0371 (4) Å
*c* = 14.4408 (5) Å
β = 105.8460 (7)°
*V* = 3365.8 (2) Å^3^
*Z* = 4
Mo *K*α radiation
μ = 0.08 mm^−1^
*T* = 100 K
0.30 × 0.30 × 0.30 mm

#### Data collection

Bruker APEXII diffractometer
35921 measured reflections
7717 independent reflections
6410 reflections with *I* > 2σ(*I*)
*R*_int_ = 0.035

#### Refinement

*R*[*F* ^2^ > 2σ(*F* ^2^)] = 0.041
*wR*(*F* ^2^) = 0.111
*S* = 1.03
7717 reflections
415 parameters
H-atom parameters constrained
Δρ_max_ = 0.35 e Å^−3^
Δρ_min_ = −0.25 e Å^−3^

### 

Data collection: *APEX2* (Bruker, 2005 ▶); cell refinement: *SAINT* (Bruker, 2005 ▶); data reduction: *SAINT*; program(s) used to solve structure: *SHELXS97* (Sheldrick, 2008 ▶); program(s) used to refine structure: *SHELXL97* (Sheldrick, 2008 ▶); molecular graphics: *X-SEED* (Barbour, 2001 ▶); software used to prepare material for publication: *publCIF* (Westrip, 2010 ▶).

## Supplementary Material

Crystal structure: contains datablocks global, I. DOI: 10.1107/S1600536811007318/lh5213sup1.cif

Structure factors: contains datablocks I. DOI: 10.1107/S1600536811007318/lh5213Isup2.hkl

Additional supplementary materials:  crystallographic information; 3D view; checkCIF report

## Figures and Tables

**Fig. 1.**
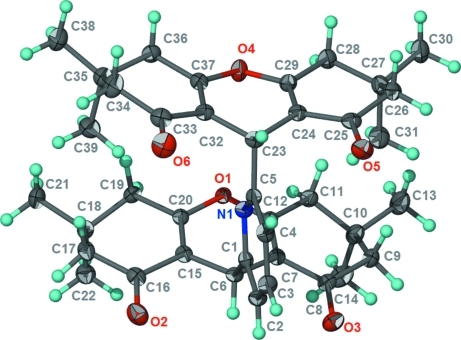
Thermal ellipsoid plot (Barbour, 2001) of C39H45NO6 at the 70% probability level; hydrogen atoms are drawn as spheres of arbitrary radius.

**Fig. 2.**
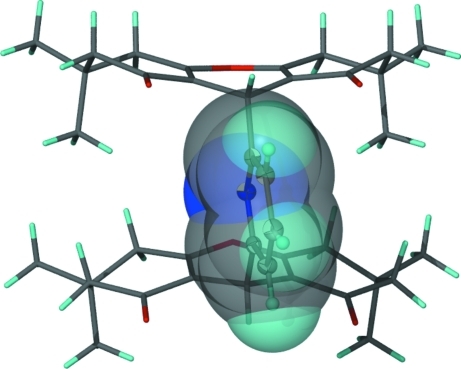
View of the molecule along the pyridyl plane. The pyridyl atoms are shown as 70% thermal ellipsoids in their van der Waals surfaces whereas the other atoms are shown in the ball-and-stick style.

## supplementary crystallographic information

### Comment

The reaction of pyridine-2,6-dicarboxaldehyde, amino-2-propanol and dimedone in ethanol yields the ethanol solvate of 4*α*-hydroxy-3,3,6,6-tetramethyl-9-[6-(3,3,6,6-tetramethyl-1,8-dioxo-2,3,4,5,6,7,8,9-octahydro-9-xanthenyl)-2-pyridyl]-2,3,4,4*α*,5,6,7,8,9,9*α*-decahydro-1,8-xanthenedione. The compound has two xanthenyldione portions but the carbon-carbon double bond of one is hydrated (Mohamed *et al.*, 2011). The title compound is that without water adding across the double bond, and was obtained by using a light different synthetic route. The pyridine ring in C_39_H_45_NO_6_ is connected to two tetramethyloctahydroxanthen-1,8-dionyl substituents at the 2- and 6-positions of the ring; the six-membered xanthenyl rings adopt flattened boat-shaped conformations (with the O and methine C atoms deviating from the plane of the other four atoms. The other six-membered rings are in sofa conformations (Fig. 1). The xanthenyl units are stacked over each other (Fig. 2).

### Experimental

A mixture of 1,2-ethanediammonium chloride (0.01 mol) and 2,6-pyridinedicarbaldehyde (0.01 mol) in aqueous ethanol (1:1) was heated for 30 minutes. To the mixture was added dimedone (0.04 mol). The mixture was heated for another 5 fhours. The solvent was evaporated to yield a pale yellow compound. This was recrystallized from ethanol in 60% yield; m.p. 473 K.

### Refinement

Carbon-bound H-atoms were placed in calculated positions [C–H 0.95 to 0.99 Å; *U*(H) 1.2 to 1.5*U*(C)] and were included in the refinement in the riding model approximation.

### Crystal data

**Table:**

C_39_H_45_NO_6_	*F*(000) = 1336
*M**_r_* = 623.76	*D*_x_ = 1.231 Mg m^−^^3^
Monoclinic, *P*2_1_/*c*	Mo *K*α radiation, λ = 0.71073 Å
Hall symbol: -P 2ybc	Cell parameters from 9997 reflections
*a* = 24.1384 (8) Å	θ = 2.2–28.3°
*b* = 10.0371 (4) Å	µ = 0.08 mm^−^^1^
*c* = 14.4408 (5) Å	*T* = 100 K
β = 105.8460 (7)°	Prism, yellow
*V* = 3365.8 (2) Å^3^	0.30 × 0.30 × 0.30 mm
*Z* = 4	

### Data collection

**Table:**

Bruker APEXII diffractometer	6410 reflections with *I* > 2σ(*I*)
Radiation source: fine-focus sealed tube	*R*_int_ = 0.035
graphite	θ_max_ = 27.5°, θ_min_ = 0.9°
φ and ω scans	*h* = −31→31
35921 measured reflections	*k* = −13→13
7717 independent reflections	*l* = −18→18

### Refinement

**Table:**

Refinement on *F*^2^	Primary atom site location: structure-invariant direct methods
Least-squares matrix: full	Secondary atom site location: difference Fourier map
*R*[*F*^2^ > 2σ(*F*^2^)] = 0.041	Hydrogen site location: inferred from neighbouring sites
*wR*(*F*^2^) = 0.111	H-atom parameters constrained
*S* = 1.03	*w* = 1/[σ^2^(*F*_o_^2^) + (0.0516*P*)^2^ + 1.4985*P*] where *P* = (*F*_o_^2^ + 2*F*_c_^2^)/3
7717 reflections	(Δ/σ)_max_ = 0.001
415 parameters	Δρ_max_ = 0.35 e Å^−^^3^
0 restraints	Δρ_min_ = −0.25 e Å^−^^3^

### Fractional atomic coordinates and isotropic or equivalent isotropic displacement parameters (Å^2^)

**Table:**

	*x*	*y*	*z*	*U*_iso_*/*U*_eq_	
O1	0.25761 (4)	0.57513 (8)	0.57197 (6)	0.01520 (18)	
O2	0.14471 (4)	0.79864 (10)	0.29720 (7)	0.0240 (2)	
O3	0.36827 (4)	0.79429 (9)	0.39499 (7)	0.0219 (2)	
O4	0.24055 (4)	0.16338 (10)	0.42959 (6)	0.0194 (2)	
O5	0.35334 (4)	0.23059 (10)	0.21320 (7)	0.0235 (2)	
O6	0.12820 (4)	0.24399 (11)	0.11215 (7)	0.0283 (2)	
N1	0.24746 (4)	0.48227 (10)	0.31079 (7)	0.0153 (2)	
C1	0.25467 (5)	0.61420 (12)	0.30329 (8)	0.0139 (2)	
C2	0.26132 (5)	0.67429 (13)	0.22082 (9)	0.0171 (2)	
H2	0.2668	0.7678	0.2187	0.020*	
C3	0.25984 (5)	0.59557 (13)	0.14138 (9)	0.0185 (3)	
H3	0.2644	0.6341	0.0838	0.022*	
C4	0.25163 (5)	0.46041 (13)	0.14736 (9)	0.0163 (2)	
H4	0.2498	0.4044	0.0936	0.020*	
C5	0.24614 (5)	0.40734 (12)	0.23306 (8)	0.0142 (2)	
C6	0.25609 (5)	0.70059 (12)	0.39089 (8)	0.0143 (2)	
H6	0.2567	0.7960	0.3712	0.017*	
C7	0.30987 (5)	0.67488 (12)	0.47187 (9)	0.0144 (2)	
C8	0.36561 (5)	0.72160 (12)	0.46172 (9)	0.0160 (2)	
C9	0.41928 (5)	0.67402 (13)	0.53518 (9)	0.0189 (3)	
H9A	0.4512	0.7362	0.5355	0.023*	
H9B	0.4300	0.5855	0.5151	0.023*	
C10	0.41313 (5)	0.66312 (13)	0.63785 (9)	0.0196 (3)	
C11	0.35893 (5)	0.58161 (13)	0.63655 (9)	0.0168 (2)	
H11A	0.3680	0.4857	0.6343	0.020*	
H11B	0.3484	0.5980	0.6972	0.020*	
C12	0.30824 (5)	0.61373 (12)	0.55352 (9)	0.0139 (2)	
C13	0.46594 (6)	0.59178 (16)	0.70204 (11)	0.0294 (3)	
H13A	0.5007	0.6430	0.7034	0.044*	
H13B	0.4690	0.5026	0.6763	0.044*	
H13C	0.4619	0.5842	0.7675	0.044*	
C14	0.40892 (6)	0.80262 (14)	0.67847 (10)	0.0260 (3)	
H14A	0.4436	0.8535	0.6789	0.039*	
H14B	0.4054	0.7951	0.7443	0.039*	
H14C	0.3750	0.8484	0.6382	0.039*	
C15	0.20391 (5)	0.68011 (12)	0.42723 (9)	0.0148 (2)	
C16	0.14805 (5)	0.73105 (13)	0.36908 (9)	0.0172 (2)	
C17	0.09520 (5)	0.69456 (13)	0.40051 (9)	0.0193 (3)	
H17A	0.0811	0.6065	0.3731	0.023*	
H17B	0.0646	0.7607	0.3737	0.023*	
C18	0.10563 (5)	0.68897 (13)	0.51000 (9)	0.0181 (3)	
C19	0.15724 (5)	0.59658 (12)	0.55268 (9)	0.0174 (2)	
H19A	0.1704	0.6102	0.6232	0.021*	
H19B	0.1446	0.5028	0.5409	0.021*	
C20	0.20658 (5)	0.62020 (12)	0.51102 (9)	0.0144 (2)	
C21	0.05265 (6)	0.63199 (15)	0.53434 (11)	0.0254 (3)	
H21A	0.0194	0.6899	0.5076	0.038*	
H21B	0.0599	0.6270	0.6044	0.038*	
H21C	0.0446	0.5426	0.5067	0.038*	
C22	0.11671 (6)	0.82998 (13)	0.55179 (10)	0.0212 (3)	
H22A	0.0832	0.8864	0.5234	0.032*	
H22B	0.1508	0.8673	0.5369	0.032*	
H22C	0.1232	0.8264	0.6218	0.032*	
C23	0.24111 (5)	0.25647 (12)	0.24168 (8)	0.0152 (2)	
H23	0.2399	0.2159	0.1779	0.018*	
C24	0.29335 (5)	0.20300 (12)	0.31524 (9)	0.0158 (2)	
C25	0.34866 (6)	0.19442 (12)	0.29151 (9)	0.0174 (3)	
C26	0.39889 (5)	0.13376 (13)	0.36653 (9)	0.0185 (3)	
H26A	0.4353	0.1653	0.3551	0.022*	
H26B	0.3975	0.0356	0.3592	0.022*	
C27	0.39860 (5)	0.16943 (13)	0.46965 (9)	0.0184 (3)	
C28	0.34085 (5)	0.12467 (13)	0.48417 (9)	0.0188 (3)	
H28A	0.3407	0.0264	0.4899	0.023*	
H28B	0.3365	0.1628	0.5451	0.023*	
C29	0.29112 (5)	0.16688 (12)	0.40344 (9)	0.0163 (2)	
C30	0.44699 (6)	0.09678 (15)	0.54235 (10)	0.0252 (3)	
H30A	0.4462	0.1207	0.6078	0.038*	
H30B	0.4841	0.1226	0.5326	0.038*	
H30C	0.4417	0.0004	0.5334	0.038*	
C31	0.40642 (6)	0.31961 (14)	0.48608 (11)	0.0253 (3)	
H31A	0.4061	0.3410	0.5522	0.038*	
H31B	0.3749	0.3669	0.4408	0.038*	
H31C	0.4432	0.3472	0.4758	0.038*	
C32	0.18784 (5)	0.21360 (12)	0.26884 (9)	0.0160 (2)	
C33	0.13235 (6)	0.20954 (13)	0.19479 (9)	0.0195 (3)	
C34	0.08132 (6)	0.15408 (15)	0.22403 (9)	0.0237 (3)	
H34A	0.0800	0.0564	0.2142	0.028*	
H34B	0.0456	0.1920	0.1811	0.028*	
C35	0.08224 (5)	0.18284 (13)	0.32849 (9)	0.0186 (3)	
C36	0.13980 (5)	0.13223 (13)	0.39325 (9)	0.0180 (3)	
H36A	0.1451	0.1668	0.4593	0.022*	
H36B	0.1388	0.0337	0.3962	0.022*	
C37	0.18971 (5)	0.17401 (12)	0.35799 (9)	0.0159 (2)	
C38	0.03288 (6)	0.10894 (15)	0.35365 (10)	0.0254 (3)	
H38A	−0.0040	0.1410	0.3124	0.038*	
H38B	0.0345	0.1253	0.4213	0.038*	
H38C	0.0365	0.0132	0.3434	0.038*	
C39	0.07642 (6)	0.33236 (14)	0.34388 (11)	0.0258 (3)	
H39A	0.0396	0.3640	0.3023	0.039*	
H39B	0.1081	0.3797	0.3278	0.039*	
H39C	0.0779	0.3491	0.4114	0.039*	

### Atomic displacement parameters (Å^2^)

**Table:**

	*U*^11^	*U*^22^	*U*^33^	*U*^12^	*U*^13^	*U*^23^
O1	0.0141 (4)	0.0163 (4)	0.0153 (4)	−0.0004 (3)	0.0043 (3)	0.0024 (3)
O2	0.0251 (5)	0.0286 (5)	0.0178 (5)	0.0065 (4)	0.0048 (4)	0.0037 (4)
O3	0.0244 (5)	0.0234 (5)	0.0190 (5)	−0.0044 (4)	0.0078 (4)	0.0021 (4)
O4	0.0159 (4)	0.0302 (5)	0.0131 (4)	0.0013 (4)	0.0055 (3)	0.0037 (4)
O5	0.0300 (5)	0.0246 (5)	0.0205 (5)	0.0050 (4)	0.0149 (4)	0.0045 (4)
O6	0.0298 (5)	0.0392 (6)	0.0140 (4)	−0.0012 (4)	0.0030 (4)	0.0029 (4)
N1	0.0178 (5)	0.0161 (5)	0.0124 (5)	0.0001 (4)	0.0049 (4)	0.0005 (4)
C1	0.0120 (5)	0.0160 (6)	0.0129 (5)	0.0011 (4)	0.0021 (4)	−0.0003 (4)
C2	0.0189 (6)	0.0157 (6)	0.0166 (6)	0.0001 (5)	0.0048 (5)	0.0031 (5)
C3	0.0205 (6)	0.0224 (6)	0.0137 (6)	0.0013 (5)	0.0066 (5)	0.0043 (5)
C4	0.0172 (6)	0.0206 (6)	0.0114 (5)	0.0016 (5)	0.0043 (5)	−0.0002 (5)
C5	0.0136 (5)	0.0159 (6)	0.0131 (5)	0.0008 (4)	0.0036 (4)	0.0008 (4)
C6	0.0168 (6)	0.0132 (5)	0.0130 (5)	0.0004 (4)	0.0044 (5)	0.0011 (4)
C7	0.0154 (6)	0.0135 (5)	0.0142 (6)	−0.0002 (4)	0.0039 (5)	−0.0024 (4)
C8	0.0194 (6)	0.0147 (6)	0.0153 (6)	−0.0020 (5)	0.0069 (5)	−0.0031 (5)
C9	0.0156 (6)	0.0203 (6)	0.0215 (6)	−0.0018 (5)	0.0060 (5)	0.0005 (5)
C10	0.0163 (6)	0.0235 (7)	0.0175 (6)	−0.0036 (5)	0.0019 (5)	0.0019 (5)
C11	0.0169 (6)	0.0183 (6)	0.0147 (6)	−0.0004 (5)	0.0035 (5)	0.0022 (5)
C12	0.0145 (6)	0.0112 (5)	0.0162 (6)	−0.0009 (4)	0.0049 (5)	−0.0025 (4)
C13	0.0180 (7)	0.0394 (8)	0.0266 (7)	−0.0042 (6)	−0.0009 (6)	0.0108 (6)
C14	0.0300 (7)	0.0274 (7)	0.0200 (7)	−0.0103 (6)	0.0057 (6)	−0.0044 (5)
C15	0.0164 (6)	0.0135 (5)	0.0144 (6)	0.0002 (4)	0.0040 (5)	−0.0034 (4)
C16	0.0199 (6)	0.0160 (6)	0.0149 (6)	0.0019 (5)	0.0033 (5)	−0.0033 (5)
C17	0.0156 (6)	0.0201 (6)	0.0205 (6)	0.0013 (5)	0.0021 (5)	−0.0028 (5)
C18	0.0158 (6)	0.0185 (6)	0.0209 (6)	0.0004 (5)	0.0066 (5)	0.0001 (5)
C19	0.0171 (6)	0.0169 (6)	0.0189 (6)	−0.0016 (5)	0.0064 (5)	0.0014 (5)
C20	0.0144 (6)	0.0125 (5)	0.0161 (6)	0.0000 (4)	0.0038 (5)	−0.0023 (4)
C21	0.0171 (6)	0.0269 (7)	0.0345 (8)	0.0006 (5)	0.0109 (6)	0.0036 (6)
C22	0.0227 (6)	0.0204 (6)	0.0218 (6)	0.0016 (5)	0.0083 (5)	−0.0011 (5)
C23	0.0208 (6)	0.0152 (6)	0.0104 (5)	0.0007 (5)	0.0057 (5)	−0.0006 (4)
C24	0.0193 (6)	0.0141 (6)	0.0151 (6)	0.0009 (5)	0.0065 (5)	−0.0006 (4)
C25	0.0239 (6)	0.0132 (6)	0.0175 (6)	0.0009 (5)	0.0095 (5)	−0.0014 (5)
C26	0.0200 (6)	0.0179 (6)	0.0207 (6)	0.0030 (5)	0.0109 (5)	0.0007 (5)
C27	0.0176 (6)	0.0203 (6)	0.0183 (6)	0.0025 (5)	0.0066 (5)	0.0006 (5)
C28	0.0187 (6)	0.0239 (6)	0.0148 (6)	0.0021 (5)	0.0063 (5)	0.0036 (5)
C29	0.0173 (6)	0.0166 (6)	0.0166 (6)	0.0004 (5)	0.0074 (5)	0.0000 (5)
C30	0.0185 (6)	0.0341 (8)	0.0235 (7)	0.0037 (6)	0.0061 (5)	0.0046 (6)
C31	0.0233 (7)	0.0233 (7)	0.0292 (7)	−0.0004 (5)	0.0072 (6)	−0.0052 (6)
C32	0.0195 (6)	0.0140 (6)	0.0146 (6)	0.0000 (5)	0.0051 (5)	−0.0011 (4)
C33	0.0235 (7)	0.0195 (6)	0.0147 (6)	−0.0002 (5)	0.0039 (5)	−0.0021 (5)
C34	0.0201 (6)	0.0320 (7)	0.0173 (6)	−0.0059 (6)	0.0022 (5)	−0.0038 (5)
C35	0.0186 (6)	0.0196 (6)	0.0174 (6)	−0.0009 (5)	0.0046 (5)	−0.0008 (5)
C36	0.0202 (6)	0.0197 (6)	0.0151 (6)	−0.0002 (5)	0.0065 (5)	0.0024 (5)
C37	0.0172 (6)	0.0152 (6)	0.0149 (6)	0.0007 (5)	0.0035 (5)	−0.0010 (5)
C38	0.0201 (7)	0.0301 (7)	0.0262 (7)	−0.0035 (6)	0.0066 (5)	0.0000 (6)
C39	0.0266 (7)	0.0212 (7)	0.0311 (8)	0.0034 (5)	0.0102 (6)	0.0001 (6)

### Geometric parameters (Å, °)

**Table:**

O1—C12	1.3757 (14)	C19—C20	1.4926 (17)
O1—C20	1.3800 (14)	C19—H19A	0.9900
O2—C16	1.2242 (16)	C19—H19B	0.9900
O3—C8	1.2242 (15)	C21—H21A	0.9800
O4—C29	1.3732 (15)	C21—H21B	0.9800
O4—C37	1.3761 (15)	C21—H21C	0.9800
O5—C25	1.2220 (15)	C22—H22A	0.9800
O6—C33	1.2200 (16)	C22—H22B	0.9800
N1—C1	1.3438 (16)	C22—H22C	0.9800
N1—C5	1.3443 (15)	C23—C32	1.5060 (17)
C1—C2	1.3829 (17)	C23—C24	1.5082 (17)
C1—C6	1.5264 (16)	C23—H23	1.0000
C2—C3	1.3853 (18)	C24—C29	1.3393 (17)
C2—H2	0.9500	C24—C25	1.4688 (17)
C3—C4	1.3771 (18)	C25—C26	1.5157 (18)
C3—H3	0.9500	C26—C27	1.5335 (17)
C4—C5	1.3865 (16)	C26—H26A	0.9900
C4—H4	0.9500	C26—H26B	0.9900
C5—C23	1.5270 (17)	C27—C30	1.5261 (18)
C6—C15	1.5051 (17)	C27—C31	1.5296 (19)
C6—C7	1.5133 (16)	C27—C28	1.5325 (17)
C6—H6	1.0000	C28—C29	1.4884 (17)
C7—C12	1.3393 (17)	C28—H28A	0.9900
C7—C8	1.4696 (17)	C28—H28B	0.9900
C8—C9	1.5103 (17)	C30—H30A	0.9800
C9—C10	1.5338 (18)	C30—H30B	0.9800
C9—H9A	0.9900	C30—H30C	0.9800
C9—H9B	0.9900	C31—H31A	0.9800
C10—C14	1.5321 (19)	C31—H31B	0.9800
C10—C13	1.5336 (18)	C31—H31C	0.9800
C10—C11	1.5387 (17)	C32—C37	1.3363 (17)
C11—C12	1.4958 (17)	C32—C33	1.4687 (17)
C11—H11A	0.9900	C33—C34	1.5129 (18)
C11—H11B	0.9900	C34—C35	1.5301 (18)
C13—H13A	0.9800	C34—H34A	0.9900
C13—H13B	0.9800	C34—H34B	0.9900
C13—H13C	0.9800	C35—C38	1.5293 (18)
C14—H14A	0.9800	C35—C39	1.5292 (18)
C14—H14B	0.9800	C35—C36	1.5341 (18)
C14—H14C	0.9800	C36—C37	1.4903 (17)
C15—C20	1.3368 (17)	C36—H36A	0.9900
C15—C16	1.4719 (17)	C36—H36B	0.9900
C16—C17	1.5111 (18)	C38—H38A	0.9800
C17—C18	1.5328 (18)	C38—H38B	0.9800
C17—H17A	0.9900	C38—H38C	0.9800
C17—H17B	0.9900	C39—H39A	0.9800
C18—C21	1.5269 (17)	C39—H39B	0.9800
C18—C22	1.5332 (18)	C39—H39C	0.9800
C18—C19	1.5403 (17)		
			
C12—O1—C20	118.01 (9)	C18—C21—H21C	109.5
C29—O4—C37	117.95 (9)	H21A—C21—H21C	109.5
C1—N1—C5	117.26 (10)	H21B—C21—H21C	109.5
N1—C1—C2	123.18 (11)	C18—C22—H22A	109.5
N1—C1—C6	117.84 (10)	C18—C22—H22B	109.5
C2—C1—C6	118.97 (11)	H22A—C22—H22B	109.5
C1—C2—C3	118.79 (12)	C18—C22—H22C	109.5
C1—C2—H2	120.6	H22A—C22—H22C	109.5
C3—C2—H2	120.6	H22B—C22—H22C	109.5
C4—C3—C2	118.78 (11)	C32—C23—C24	108.91 (10)
C4—C3—H3	120.6	C32—C23—C5	113.40 (10)
C2—C3—H3	120.6	C24—C23—C5	109.91 (10)
C3—C4—C5	119.01 (11)	C32—C23—H23	108.2
C3—C4—H4	120.5	C24—C23—H23	108.2
C5—C4—H4	120.5	C5—C23—H23	108.2
N1—C5—C4	122.97 (11)	C29—C24—C25	118.76 (11)
N1—C5—C23	118.10 (10)	C29—C24—C23	121.54 (11)
C4—C5—C23	118.87 (11)	C25—C24—C23	119.66 (10)
C15—C6—C7	109.28 (10)	O5—C25—C24	121.22 (12)
C15—C6—C1	112.32 (10)	O5—C25—C26	121.50 (11)
C7—C6—C1	111.35 (10)	C24—C25—C26	117.23 (10)
C15—C6—H6	107.9	C25—C26—C27	112.66 (10)
C7—C6—H6	107.9	C25—C26—H26A	109.1
C1—C6—H6	107.9	C27—C26—H26A	109.1
C12—C7—C8	118.80 (11)	C25—C26—H26B	109.1
C12—C7—C6	122.23 (11)	C27—C26—H26B	109.1
C8—C7—C6	118.95 (10)	H26A—C26—H26B	107.8
O3—C8—C7	120.99 (11)	C30—C27—C31	108.98 (11)
O3—C8—C9	121.47 (11)	C30—C27—C28	108.65 (11)
C7—C8—C9	117.52 (10)	C31—C27—C28	110.25 (11)
C8—C9—C10	114.25 (10)	C30—C27—C26	110.53 (11)
C8—C9—H9A	108.7	C31—C27—C26	110.34 (11)
C10—C9—H9A	108.7	C28—C27—C26	108.06 (10)
C8—C9—H9B	108.7	C29—C28—C27	112.24 (10)
C10—C9—H9B	108.7	C29—C28—H28A	109.2
H9A—C9—H9B	107.6	C27—C28—H28A	109.2
C14—C10—C13	109.05 (11)	C29—C28—H28B	109.2
C14—C10—C9	109.82 (11)	C27—C28—H28B	109.2
C13—C10—C9	109.49 (11)	H28A—C28—H28B	107.9
C14—C10—C11	110.21 (11)	C24—C29—O4	122.55 (11)
C13—C10—C11	108.83 (11)	C24—C29—C28	126.06 (11)
C9—C10—C11	109.42 (10)	O4—C29—C28	111.39 (10)
C12—C11—C10	113.69 (10)	C27—C30—H30A	109.5
C12—C11—H11A	108.8	C27—C30—H30B	109.5
C10—C11—H11A	108.8	H30A—C30—H30B	109.5
C12—C11—H11B	108.8	C27—C30—H30C	109.5
C10—C11—H11B	108.8	H30A—C30—H30C	109.5
H11A—C11—H11B	107.7	H30B—C30—H30C	109.5
C7—C12—O1	122.73 (11)	C27—C31—H31A	109.5
C7—C12—C11	126.18 (11)	C27—C31—H31B	109.5
O1—C12—C11	111.07 (10)	H31A—C31—H31B	109.5
C10—C13—H13A	109.5	C27—C31—H31C	109.5
C10—C13—H13B	109.5	H31A—C31—H31C	109.5
H13A—C13—H13B	109.5	H31B—C31—H31C	109.5
C10—C13—H13C	109.5	C37—C32—C33	118.51 (11)
H13A—C13—H13C	109.5	C37—C32—C23	121.95 (11)
H13B—C13—H13C	109.5	C33—C32—C23	119.49 (11)
C10—C14—H14A	109.5	O6—C33—C32	121.28 (12)
C10—C14—H14B	109.5	O6—C33—C34	121.44 (12)
H14A—C14—H14B	109.5	C32—C33—C34	117.21 (11)
C10—C14—H14C	109.5	C33—C34—C35	114.32 (11)
H14A—C14—H14C	109.5	C33—C34—H34A	108.7
H14B—C14—H14C	109.5	C35—C34—H34A	108.7
C20—C15—C16	118.88 (11)	C33—C34—H34B	108.7
C20—C15—C6	122.60 (11)	C35—C34—H34B	108.7
C16—C15—C6	118.50 (11)	H34A—C34—H34B	107.6
O2—C16—C15	120.94 (12)	C38—C35—C39	109.44 (11)
O2—C16—C17	121.57 (11)	C38—C35—C34	109.89 (11)
C15—C16—C17	117.48 (11)	C39—C35—C34	110.57 (11)
C16—C17—C18	113.85 (10)	C38—C35—C36	109.23 (11)
C16—C17—H17A	108.8	C39—C35—C36	109.89 (11)
C18—C17—H17A	108.8	C34—C35—C36	107.79 (10)
C16—C17—H17B	108.8	C37—C36—C35	112.36 (10)
C18—C17—H17B	108.8	C37—C36—H36A	109.1
H17A—C17—H17B	107.7	C35—C36—H36A	109.1
C21—C18—C17	109.83 (11)	C37—C36—H36B	109.1
C21—C18—C22	108.80 (11)	C35—C36—H36B	109.1
C17—C18—C22	109.51 (11)	H36A—C36—H36B	107.9
C21—C18—C19	108.62 (10)	C32—C37—O4	122.38 (11)
C17—C18—C19	108.78 (10)	C32—C37—C36	126.52 (11)
C22—C18—C19	111.30 (10)	O4—C37—C36	111.08 (10)
C20—C19—C18	112.98 (10)	C35—C38—H38A	109.5
C20—C19—H19A	109.0	C35—C38—H38B	109.5
C18—C19—H19A	109.0	H38A—C38—H38B	109.5
C20—C19—H19B	109.0	C35—C38—H38C	109.5
C18—C19—H19B	109.0	H38A—C38—H38C	109.5
H19A—C19—H19B	107.8	H38B—C38—H38C	109.5
C15—C20—O1	122.60 (11)	C35—C39—H39A	109.5
C15—C20—C19	126.13 (11)	C35—C39—H39B	109.5
O1—C20—C19	111.25 (10)	H39A—C39—H39B	109.5
C18—C21—H21A	109.5	C35—C39—H39C	109.5
C18—C21—H21B	109.5	H39A—C39—H39C	109.5
H21A—C21—H21B	109.5	H39B—C39—H39C	109.5
			
C5—N1—C1—C2	−0.78 (18)	C6—C15—C20—C19	179.61 (11)
C5—N1—C1—C6	179.85 (10)	C12—O1—C20—C15	11.41 (16)
N1—C1—C2—C3	0.82 (19)	C12—O1—C20—C19	−166.90 (10)
C6—C1—C2—C3	−179.82 (11)	C18—C19—C20—C15	−20.41 (18)
C1—C2—C3—C4	0.18 (18)	C18—C19—C20—O1	157.83 (10)
C2—C3—C4—C5	−1.12 (18)	N1—C5—C23—C32	58.96 (14)
C1—N1—C5—C4	−0.24 (17)	C4—C5—C23—C32	−123.78 (12)
C1—N1—C5—C23	176.90 (10)	N1—C5—C23—C24	−63.20 (14)
C3—C4—C5—N1	1.19 (19)	C4—C5—C23—C24	114.06 (12)
C3—C4—C5—C23	−175.93 (11)	C32—C23—C24—C29	−20.88 (16)
N1—C1—C6—C15	−54.14 (14)	C5—C23—C24—C29	103.91 (13)
C2—C1—C6—C15	126.46 (12)	C32—C23—C24—C25	161.34 (10)
N1—C1—C6—C7	68.80 (14)	C5—C23—C24—C25	−73.87 (13)
C2—C1—C6—C7	−110.60 (12)	C29—C24—C25—O5	−177.42 (12)
C15—C6—C7—C12	14.18 (16)	C23—C24—C25—O5	0.42 (18)
C1—C6—C7—C12	−110.48 (13)	C29—C24—C25—C26	5.01 (17)
C15—C6—C7—C8	−164.21 (10)	C23—C24—C25—C26	−177.15 (11)
C1—C6—C7—C8	71.13 (13)	O5—C25—C26—C27	146.24 (12)
C12—C7—C8—O3	−169.42 (12)	C24—C25—C26—C27	−36.19 (15)
C6—C7—C8—O3	9.03 (17)	C25—C26—C27—C30	175.29 (11)
C12—C7—C8—C9	11.97 (17)	C25—C26—C27—C31	−64.09 (14)
C6—C7—C8—C9	−169.59 (10)	C25—C26—C27—C28	56.52 (14)
O3—C8—C9—C10	143.94 (12)	C30—C27—C28—C29	−167.43 (11)
C7—C8—C9—C10	−37.46 (15)	C31—C27—C28—C29	73.20 (14)
C8—C9—C10—C14	−70.02 (14)	C26—C27—C28—C29	−47.46 (14)
C8—C9—C10—C13	170.28 (11)	C25—C24—C29—O4	−175.58 (11)
C8—C9—C10—C11	51.08 (14)	C23—C24—C29—O4	6.62 (19)
C14—C10—C11—C12	79.67 (13)	C25—C24—C29—C28	3.67 (19)
C13—C10—C11—C12	−160.79 (11)	C23—C24—C29—C28	−174.13 (12)
C9—C10—C11—C12	−41.18 (14)	C37—O4—C29—C24	11.94 (18)
C8—C7—C12—O1	175.34 (10)	C37—O4—C29—C28	−167.41 (10)
C6—C7—C12—O1	−3.05 (18)	C27—C28—C29—C24	19.21 (18)
C8—C7—C12—C11	−2.85 (19)	C27—C28—C29—O4	−161.47 (10)
C6—C7—C12—C11	178.76 (11)	C24—C23—C32—C37	19.29 (16)
C20—O1—C12—C7	−10.61 (16)	C5—C23—C32—C37	−103.42 (14)
C20—O1—C12—C11	167.82 (10)	C24—C23—C32—C33	−158.13 (11)
C10—C11—C12—C7	18.90 (18)	C5—C23—C32—C33	79.16 (14)
C10—C11—C12—O1	−159.47 (10)	C37—C32—C33—O6	−179.69 (13)
C7—C6—C15—C20	−13.46 (16)	C23—C32—C33—O6	−2.18 (19)
C1—C6—C15—C20	110.63 (13)	C37—C32—C33—C34	−2.84 (18)
C7—C6—C15—C16	165.04 (10)	C23—C32—C33—C34	174.67 (11)
C1—C6—C15—C16	−70.86 (13)	O6—C33—C34—C35	−149.70 (13)
C20—C15—C16—O2	172.25 (12)	C32—C33—C34—C35	33.45 (17)
C6—C15—C16—O2	−6.31 (17)	C33—C34—C35—C38	−173.37 (12)
C20—C15—C16—C17	−8.86 (17)	C33—C34—C35—C39	65.70 (15)
C6—C15—C16—C17	172.58 (10)	C33—C34—C35—C36	−54.43 (15)
O2—C16—C17—C18	−144.91 (12)	C38—C35—C36—C37	166.01 (11)
C15—C16—C17—C18	36.21 (15)	C39—C35—C36—C37	−73.91 (14)
C16—C17—C18—C21	−171.76 (11)	C34—C35—C36—C37	46.65 (14)
C16—C17—C18—C22	68.82 (13)	C33—C32—C37—O4	174.15 (11)
C16—C17—C18—C19	−53.01 (14)	C23—C32—C37—O4	−3.29 (19)
C21—C18—C19—C20	163.90 (11)	C33—C32—C37—C36	−3.85 (19)
C17—C18—C19—C20	44.39 (14)	C23—C32—C37—C36	178.71 (11)
C22—C18—C19—C20	−76.35 (13)	C29—O4—C37—C32	−13.67 (17)
C16—C15—C20—O1	−176.94 (10)	C29—O4—C37—C36	164.61 (10)
C6—C15—C20—O1	1.56 (18)	C35—C36—C37—C32	−19.89 (18)
C16—C15—C20—C19	1.11 (19)	C35—C36—C37—O4	161.92 (10)
